# Complete Genome Sequence of the Porcine Epidemic Diarrhea Virus Variant CH/HNYF/2014

**DOI:** 10.1128/genomeA.01486-15

**Published:** 2015-12-17

**Authors:** Renfeng Li, Xiangqin Tian, Songlin Qiao, Junqing Guo, Weitao Xie, Gaiping Zhang

**Affiliations:** a College of Veterinary Medicine, Henan Institute of Science and Technology, Xinxiang, Henan, China; b Key Laboratory of Animal Immunology of the Ministry of Agriculture, Henan Provincial Key Laboratory of Animal Immunology, Henan Academy of Agricultural Sciences, Zhengzhou, Henan, China; c Medical Research Center, Xinxiang Medical University, Xinxiang, China; d College of Animal Science and Veterinary Medicine, Henan Agricultural University, Zhengzhou, China

## Abstract

Sow’s milk is a potential route for the vertical transmission of porcine epidemic diarrhea virus (PEDV) from sow to suckling piglet. We report here the complete genome sequence of PEDV strain CH/HNYF/2014, which was isolated from milk samples**.** This information provides further understanding of the transmission mechanisms and genetic diversity of PEDV.

## GENOME ANNOUNCEMENT

Porcine epidemic diarrhea virus (PEDV) is the causative agent of porcine epidemic diarrhea (PED), an acute and highly contagious enteric disease characterized by watery diarrhea and vomiting. PEDV was first recognized as the causative agent of PED in Europe in 1971 (1), and it has occurred on pig farms in many Asian countries and more recently in the United States and Germany (2, 3). It was previously reported that PEDV can be transmitted vertically from sow to suckling piglet via milk (4). Here, we report the complete genome sequence of a variant PEDV strain (CH/HNYF/2014) isolated from sow milk samples.

In this study, PEDV strain CH/HNYF/2014 was isolated from milk samples from an immunized infected sow on a pig breeding farm in Henan Province, China. To determine the complete genome sequence of the PEDV isolate CH/HNYF/2014, 12 sets of primers based on the CV777 strain were designed to cover the full-length genome of PEDV. The complete genome of CH/HNYF/2014 is 27,996 nucleotides (nt) in length, excluding the poly(A) tail, and the genome organization is as follows: a 5′ untranslated region (UTR) of 292 nt (nt 1 to 292), an open reading frame 1a (ORF1a) and ORF1b encoding a replicase of 20,345 nt (nt 293 to 20637), a spike (S) gene of 4,141 nt (nt 20634 to 24774), an ORF3 of 666 nt (nt 24773 to 25438), an envelope (E) gene of 219 nt (nt 25419 to 25637), a membrane (M) gene of 681 nt (nt 25645 to 26325), a nucleocapsid (N) gene of 1,326 nt (nt 26337 to 27662), and a 3′ UTR (nt 27663 to 27996).

The complete genome sequence of CH/HNYF/2014 shares 98.4%, 98.2%, 97.4%, 97.1%, and 97.0% nucleotide sequence identity with CH/FJND-3/2011, LC, DR13, CH/S, and the vaccine strain CV777, respectively (5–9). It exhibits 98.3% and 98.5% identity with the genome of the U.S. strain USA/Iowa96/2013 and the German strain GER/L00719/2014, respectively (2, 10). Of note, the CH/HNYF/2014 strain possesses three remarkable deletion regions compared to the CH/ZMDZY/11 strain (nt 20808 to 20819, nt 20841 to 20843, and nt 21051 to 21056) in the S gene, resulting in a continuous 4-amino-acid deletion (^59^QGVN^62^), a single-amino-acid deletion (^70^R), and a continuous 2-amino-acid deletion (^140^ND^141^). These deletion sites are mainly in the S1 domain of the S protein of CH/HNYA/2014. Further, the sequence analysis shows that there is a 30-nt deletion (nt 25058 to 25066, nt 25080 to 25088, and nt 25525 to 25536) in the ORF3 gene, which is similar to the vaccine strain CV777. Overall, we observed a high nucleotide identity of 99.3% with the variant strain CH/ZMDZY/11, which has become prevalent in Henan Province of China. These findings indicate that CH/HNYF/2014 may represent a recombinant of the PEDV vaccine strain with the current field strain.

### Nucleotide sequence accession number.

The genome sequence of PEDV strain CH/HNYF/2014 has been deposited in GenBank under the accession number KP890336.
